# An Evaluation of Probability of Adequate Nutrient Intake (PANDiet) Scores as a Diet Quality Metric in Irish National Food Consumption Data

**DOI:** 10.3390/nu14050994

**Published:** 2022-02-26

**Authors:** Laura B. Kirwan, Janette Walton, Albert Flynn, Anne P. Nugent, Breige A. McNulty

**Affiliations:** 1 Institute of Food and Health, University College Dublin, Belfield, D04 V1W8 Dublin, Ireland; laura.kirwan@ucdconnect.ie (L.B.K.); a.nugent@qub.ac.uk (A.P.N.); 2 Department of Biological Sciences, Munster Technological University, T12 P928 Cork, Ireland; janette.walton@mtu.ie; 3 School of Food and Nutritional Sciences, University College Cork, T12 K8AF Cork, Ireland; a.flynn@ucc.ie; 4 School of Biological Sciences, Institute for Global Food Security, Queens University Belfast, Belfast BT7 1NN, UK

**Keywords:** PANDiet, nutrient scores, diet quality, diet quality index, nutritional adequacy

## Abstract

Identifying reliable metrics which measure the quality of a diet to promote nutrient adequacy and long-term health is an important step in the development of a sustainable food system. The Probability of Adequate Nutrient Intake (PANDiet) scoring system has been used as a measure of dietary quality in interdisciplinary research in recent years. The aim of the current study is to apply the PANDiet scoring system, and to assess the validity of the score as a metric of nutritional adequacy, within the Irish population. The Irish National Adult Nutrition Survey is a representative database with detailed data on nutrient intakes (18–90 years; *n* = 1051 valid-reporters; 2008–2010) and biofluid analytes (blood *n* = 786; urine *n* = 778). The PANDiet scoring system was expanded to include seven macronutrients, twelve micronutrients, nine minerals, and total energy using an established methodology. PANDiet scores were assessed against the Alternate Healthy Eating Index (AHEI) and Alternate Mediterranean Diet (aMED) food-based scores. The average score for the population (μ) was 63.69 ± 0.23 and ranged from 38.27 to 89.74. Higher PANDiet scores were significantly associated with males, higher educated participants, non-smokers, and low-energy-dense diets (*p* < 0.001). Females between the ages of 18 and 35 had a significantly lower nutrient adequacy score (μ 59.17). PANDiet scores were significantly correlated with serum folate, riboflavin status, serum vitamin D (*p* < 0.05) and with AHEI and aMED scores (R_s_ 0.45 and 0.43, *p* < 0.0001). The nutritional contribution of food groups varied between genders and low, moderate, and high nutritional adequacy groups. The PANDiet scoring system facilitated a detailed analysis of nutritional adequacy across sub-groups of the population, and is a comprehensive and valid diet quality metric in Irish databases.

## 1. Introduction

Transforming food systems to be healthier and more sustainable has become a focus of international research and policy in recent years. This transformation has been identified as a complex global challenge, as it requires co-ordination between multiple actors from different disciplines. Significant dietary changes in the coming years are required in order to achieve future food demands while respecting planetary boundaries [1,2]. Identifying reliable metrics which measure the quality of a diet to promote nutrient adequacy and long-term health has been identified as an important step in guiding this dietary transition [3,4].

Diet quality indexes (DQIs) provide a simple metric to quantify the quality of an individual’s diet relative to food-based dietary guidelines (FBDG) or specific nutrient recommendations [5]. Input data used to calculate scores are typically taken from food consumption databases or food frequency questionnaires (FFQs). DQIs can be invaluable as they improve the interoperability of large datasets, and can be used to identify synergies and trade-offs that exist within and between disciplines. DQIs have been demonstrated to have good predictive ability for all-cause and non-communicable disease risk when used with non-invasive common risk factors [6]. That said, developing a DQI is complicated and many inconsistencies across DQIs exist, particularly relating to validation and the foods and/or nutrients included [4]. This presents a risk of inadequate nutrition metrics being misleading and misrepresentative of actual diet and nutritional status [7]. It is therefore imperative that scoring systems are critically evaluated before use, and, more specifically, the comparability, reliability, reproducibility, sensitivity, and specificity of scores and their association with long-term health should be considered [4,8,9,10].

Although over 80 DQIs have been previously identified in the literature [4], only two scores have been applied across multiple continents: the World Health Organisation (WHO) indicators for infants and young children [11] and the Probability of Adequate Nutrient Intake (PANDiet) scoring system [4]. PANDiet scores range between 0 and 100 and are calculated for individuals in a food consumption database based on usual intakes, with a higher score indicating a higher probability of meeting nutrient recommendations, and thus, a better diet quality [12]. The PANDiet scoring system covers a wide range of nutrients and has been applied to a number of databases globally, including Australia [13], France [14,15,16,17,18,19,20,21,22], Germany [23,24], Iran [25], Italy [24], the Netherlands [10], the UK [12,26], and the US [12] across various population groups, with the use of nutrient recommendations as constraints facilitating a cross-country comparison of results [12]. PANDiet scores provide insight into nutritional adequacy at both the population and individual level [12,27], and have been used in a clinical setting as a measure of diet quality [28,29].

The PANDiet score has also emerged as a metric of diet quality in inter-disciplinary research in recent years, particularly relating to sustainable diets [16,17,18,19]. Nutrient based-scoring systems may be particularly valuable as organisations work towards standardised and harmonised international food consumption databases [30,31] and the scope of organisations expand into assessing environmental impact [32]. As nutrient-based scores are comprehensive, progressive, and inclusive of dietary preferences, they may, therefore, become an important measure of diet quality in international multi-disciplinary research.

In Ireland, two food-based DQIs have been used in the Irish National Adult Nutrition Survey (NANS) as a measure of diet quality: the Alternate Healthy Eating Index (AHEI), and the Alternate Mediterranean Diet score (aMED) [33,34,35]. As nutritional value can significantly vary within food groups, the use of a combination of food- and nutrient-based DQIs has been recommended in order to prevent the oversight of important nutrients [12]. As dietary patterns have not shown to be static in Ireland [35], and micronutrient deficiencies in sub-groups of the population remain concerning [36,37], the use of the PANDiet scoring system may be useful for monitoring changes in nutrient adequacy over time across food consumption databases [21,22,23,24,26,27], and may facilitate the assessment of additional factors, such as environmental impact or dietary cost [14,16,17,18,19,20,22]. The current study aimed to apply an expanded PANDiet score to individuals in the Irish National Adult Nutrition Survey (NANS), and to evaluate these scores for use as a measure of diet quality.

## 2. Materials and Methods

### 2.1. Study Sample

Analyses were performed using data from the National Adult Nutrition Survey (NANS), which was a cross-sectional study carried out between 2008 and 2010 to compile information on adults between the ages of 18 and 90 years in Ireland (*n* = 1500; men: *n* = 760; women: *n* = 740). This database is considered representative of the Irish population with respect to sex, age, location, social class, and geographical location [38]. In summary, data were collected on food and beverage consumption, anthropometric measurements, and several biomarkers of nutritional status and metabolic health. Information on socio-demographic factors, food choice, physical activity levels, and lifestyle data were self-reported in questionnaires. Ethical approval was obtained from the University College Cork Clinical Research Ethics Committee of the Cork Teaching Hospitals (ECM 3 (p) 4 September 2008). Further information and a detailed methodology are available at www.iuna.net (last accessed on 16 September 2021). In the current analysis, 1051 valid reporters were included, with the Goldberg cut-off of 1.1 energy intake/basal metabolic rate ratio applied to ascertain reporting status [39,40].

### 2.2. Food Consumption Data

Information on food and beverage consumption was collected using a consecutive four-day semi-weighed food diary. Participants recorded detailed information on the amount and types of all foods and beverages consumed over the recording period, in addition to cooking methods, brand names, and recipes. Data on a total number of 2319 food and beverage items were collected. Nutrient intakes were calculated using WISP© software version 3.0 (Tinuviel Software, Anglesey, UK), based on data from McCance and Widdowson’s The Composition of Foods (5th and 6th editions), supplemented with Irish food codes. Food codes were divided into 33 food groups representative of the overall diet, which have previously been used in NANS research [33]. Food consumption data collection was spread evenly across seasons, weeks, and weekdays to ensure food consumption data was representative of habitual dietary intake.

### 2.3. Anthropometric Measurements and Socio-Demographic Factors

Anthropometric measurements, including height, weight, hip and waist circumferences, and a body composition assessment were recorded according to a defined protocol, with measurements obtained during the dietary assessment period in the participants’ homes [41]. Measurements were taken after voiding, while the participant was barefoot. Height (cm) was measured using a Leicester portable height measure (Chasmores Ltd., London, UK), and weight (kg) and body fat percentage were measured using a Tanita body composition analyser BC-420MA (Tanita Ltd., Tokyo, Japan) [41]. BMI was calculated as kg/m^2^. Three questionnaires were completed by participants in the survey, which included self-reported information on sex, age, smoking status, education status, and employment information. Self-reported information on employment was used to derive social class [41].

### 2.4. Total Estimated Energy Calculations

Total estimated energy (TEE) requirements were calculated to penalise energy-under and energy-over consumers in the PANDiet scoring system. The EPIC Physical Activity Questionnaire was used to record information on physical activity in the NANS survey [42]. Activity intensity units, referred to as metabolic equivalents (METs), were calculated and converted to minutes/week. Basal energy expenditure (BEE), physical activity level (PAL) scores, and physical activity categories (PAC) were estimated. Gender-specific TEE values were calculated, and energy intake/TEE ratios were assigned [43]. Where variables such as height, weight, and METs were unavailable (*n* = 87, 95, and 92, respectively), mean or median values by gender were used as substitute values, depending on if the data were normally distributed. Data used in the TEE calculation are outlined in Appendix A, Table A1.

### 2.5. Biofluid Data Collection and Analyses

Blood and urine samples were collected from a subset of consenting participants (blood *n* = 786; urine *n* = 778), and analysed for several markers of nutritional status and metabolic health. Blood samples were analysed for riboflavin status using erythrocyte glutathione reductase activation coefficient (EGRac), vitamin B6 status was measured using plasma pyridoxal phosphate, and serum folate and serum cobalamin were measured using microbiological assays. Concentrations of total 25-Hydroxy Vitamin D in serum samples were measured using an ELISA (OCTEIAw 25-Hydroxy Vitamin D, Immuno Diagnostic Systems Limited). Serum calcium, urinary creatinine, urinary sodium, and urinary potassium were measured using Daytona RX Clinical Analyser. Urinary sodium and potassium were corrected using mean spot urine sample concentrations for gender-specific 24-h urine volume estimations [44]. Detailed methodologies on biofluid collection, quality control, and analyses in the NANS are described elsewhere [41,45,46,47].

### 2.6. PANDiet Score Calculation

PANDiet scores were estimated for the NANS database using RStudio version 4.1.1 statistical software (RStudio IDE, Vienna, Austria). The methodology for calculating the PANDiet scores was taken from a publication by Verger et al. [12,48], and was expanded to include free sugars, iodine, pantothenic acid, biotin, and total energy. PANDiet scores consider the length of reporting period, the mean intake, day-to-day intake variability, nutrient reference value co-efficient of variance (CV), and inter-individual variability. European nutrient reference values were used as cut-off points (Appendix A, Table A2) [49,50,51]. Where nutrient reference values were weight-dependent, the mean weight for the same gender was used as a default value for unknown values. Adequacy sub-scores were calculated for 26 nutrients: protein; carbohydrate; fibre; total fat; polyunsaturated fatty acids (PUFA); vitamins A, B6, C, D, E; biotin; cobalamin; folate; niacin; pantothenic acid; riboflavin; thiamin; calcium; iodine; iron; magnesium; phosphorus; potassium; selenium; sodium; and zinc. Penalty scores of 0 were allocated where intakes exceeded upper limits for 14 nutrients: vitamins A, B6, C, D, E; calcium; folate; iodine; iron; magnesium; niacin; phosphorus; selenium; and zinc. Moderation sub-scores were calculated for 7 nutrients: protein, carbohydrate, fat, free sugars, saturated fatty acids (SFA), sodium, and total energy (kcal). The energy moderation sub-score was calculated by assigning 1 to individuals within 5% of their TEE, and 0.8, 0.6, 0.4, 0.2, and 0 scores at 20% intervals where individuals were at least 5% under or above TEE values (Appendix A, Table A3). PANDiet scores were calculated as the equally-weighted mean of the adequacy and moderation sub-scores.

### 2.7. PANDiet Score Validation

PANDiet scores were validated according to a previous methodology using content and construct validity [12], which was adjusted to data available in the NANS. Total energy density of the diet was calculated by dividing the average total energy intake (kcal) from food by the total weight of the reported food intake (g/day) [12].The relationship between PANDiet scores (dependent variable) and variables including sex, age, diet energy density, smoking status, socio economic status, education status, blood and urinary biomarkers, and food group intake (independent variables) were assessed using simple linear models, and in a multivariate model, adjusting for confounding where appropriate. *p*-value < 0.05 were considered significant. Spearman’s correlations were used to assess if the PANDiet score aligned with other dietary quality scores previously used in the NANS: the Alternate Healthy Eating Index (AHEI) and the Alternate Mediterranean Diet score (aMED), which assign diet quality scores based on food group intake [33,34,35].

### 2.8. Statistical Analyses

The statistical software package RStudio version 4.1.1 was used for all analyses in the present study. The distribution of the PANDiet scores was described using elemental statistics. Continuous variables are presented as mean ± standard error of the mean (SEM). As PANDiet scores were not normally distributed, correlation coefficients between food intakes, sub-scores, and energy intake were assessed using Spearman’s correlations. Multiple linear regressions were used to assess the relationship of PANDiet scores with demographic factors, biofluid data, and food group intakes, with corrections for confounding. Individuals were grouped into three tertiles: low, moderate, and high nutritional adequacy groups. Food group intakes and PANDiet sub-scores across groups were assessed using analysis of covariance (ANCOVA) with Bonferroni corrections for multiple comparisons, with *p* < 0.05 considered significant.

## 3. Results

PANDiet scores were calculated for 1051 participants (females *n* = 528; males *n* = 523) between the ages of 18 and 90 years in the Irish population. Participants were nationally representative relative to demographics in terms of the urban–rural divide, age group, sex, and social class, according to 2006 census data [38].

### 3.1. PANDiet Sub-Scores

**Figure 1:**
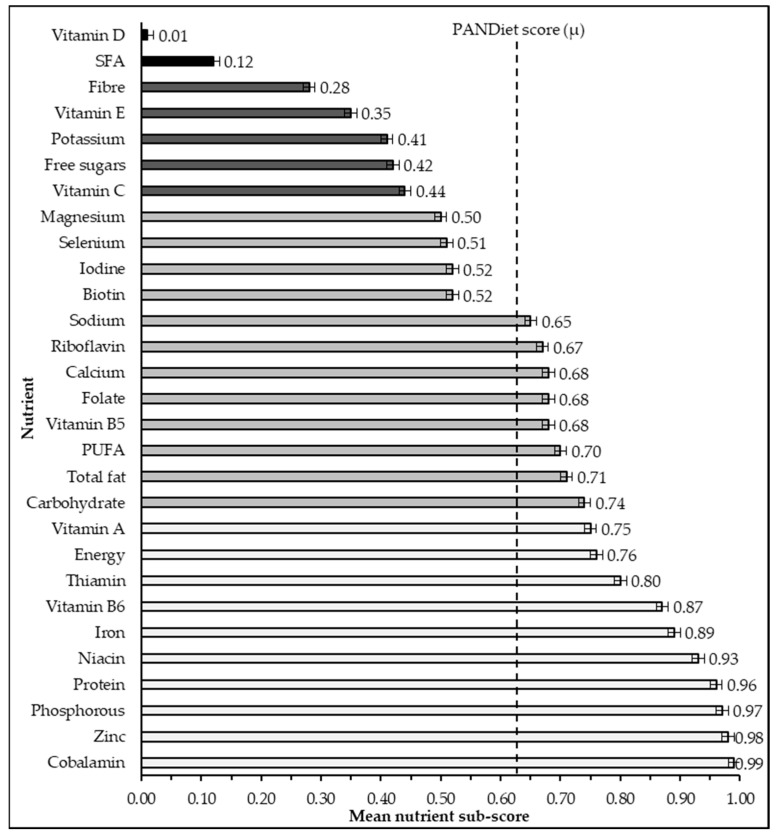
Nutrient sub-scores which form the probability of adequate nutrient intake (PANDiet) scores at the population level. Nutrient sub-scores are calculated as probability individuals will meet the nutrient recommendations outlined in Table A2. Values are shown as population mean score ± std. error. SFA = saturated fatty acids. PUFA = polyunsaturated fatty acids. All sub-scores were significantly related to PANDiet scores (*p* < 0.05), apart from PUFA (*p* = 0.05). Vertical line indicates the mean PANDiet score (63.69), scaled between 0–1 in this graph for context.

Two nutrient sub-scores had less than 25% probability of meeting recommendations: vitamin D and SFA. Four nutrients had between 25 and 50% PANDiet scores (PS): fibre, vitamin E, potassium, free sugars, and vitamin C. Eleven nutrients had between 50% and 75%: magnesium, iodine, biotin, sodium, cobalamin, calcium, folate, pantothenic acid, selenium, PUFA, fat, and carbohydrate. The remaining ten nutrients had a score over 75%: vitamin A, thiamin, total energy, folate, iron, niacin, protein, zinc, phosphorus, and cobalamin (Figure 1). Correlation with PANDiet scores was stronger for the adequacy sub-score than the moderation sub-score (0.71 and 0.46, respectively, both *p* < 0.001). Correlation was significant for all sub-scores and PANDiet scores (*p* < 0.05), apart from PUFA (*p* = 0.05). Inter-correlations between sub-scores and the PANDiet score ranged from RS −0.06 to 0.62 (Table 1).

### 3.2. PANDiet Scores

**Figure 2:**
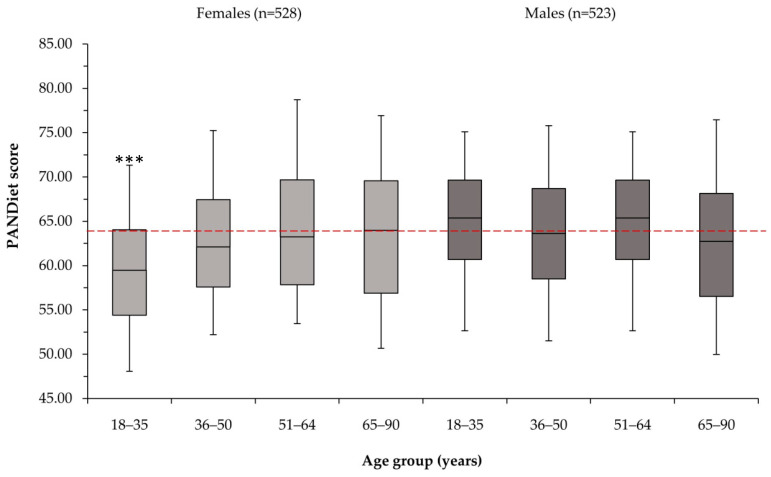
PANDiet scores by gender and age group. Horizontal dashed line indicates population median PANDiet score (63.69). Values shown as median, 50% interquartile range, and whiskers as 5 and 95% confidence intervals. *** significantly different at the <0.001 level, assessed using ANCOVA with Bonferroni post-hoc corrections for multiple comparisons.

The average PANDiet score was 63.69 ± 0.23 (range 38.27–89.74). PANDiet scores were found to be correlated at a low level to energy intake (R_s_ 0.24, *p* < 0.0001), and were approximately normally distributed (skew 0.06, kurtosis 0.23). PANDiet scores were 62.6 ± 0.34 for females, and 64.7 ± 0.30 for males (mean ± SEM). The lowest PS was found in females between the ages of 18 and 35 years (μ 59.90), followed by females from 36 to 50 years (μ 63.30), and older males between 65 and 90 years (μ 63.50). For males, no significant difference was found across age groups (*p* > 0.05). For females, a significant difference was found between the 18–35 age group and all older age groups, but no significant difference was found in nutritional adequacy in those over 35 years (*p* > 0.05) (Figure 2).

### 3.3. Nutritional Adequacy, Demographic Factors, and Nutrition Biomarkers

PANDiet scores were significantly correlated with the Alternative Healthy Eating Index (AHEI) and Alternative Mediterranean Diet (aMED) scores (R_s_ 0.45 and 0.43, respectively, both *p* < 0.0001). Participants with a higher PANDiet score were more likely to be male, be non-smokers, have a higher-education level, and have low-energy-dense diets (all *p* < 0.05). No relationship between nutritional adequacy and age or social class was found (*p* > 0.05). In the subset analysis, PANDiet scores were correlated with serum folate, riboflavin status, and serum vitamin D (*p* < 0.05), but not with serum calcium, serum ferritin, vitamin B6 status, serum vitamin B12 urinary creatinine, urinary potassium, or urinary sodium (*p* > 0.05) (Table 2).

### 3.4. Differences in Nutritional Adequacy by Gender and Age Group

When differences in PS scores were investigated between age groups for females and males, no difference was found for twelve nutrient sub-scores across groups, namely: carbohydrate, niacin, pantothenic acid, phosphorous, protein, riboflavin, SFA, thiamin, total energy, total fat, vitamin B6, and vitamin E. Significant differences were found for at least one age group for iron, selenium, and vitamin A for both genders. Scores for free sugars, fibre, folate, iodine, potassium, PUFA, and vitamin C differed between age groups for females only, and scores for biotin, calcium, cobalamin, magnesium, sodium, vitamin D, and zinc differed between age groups for males only.

**Figure 3:**
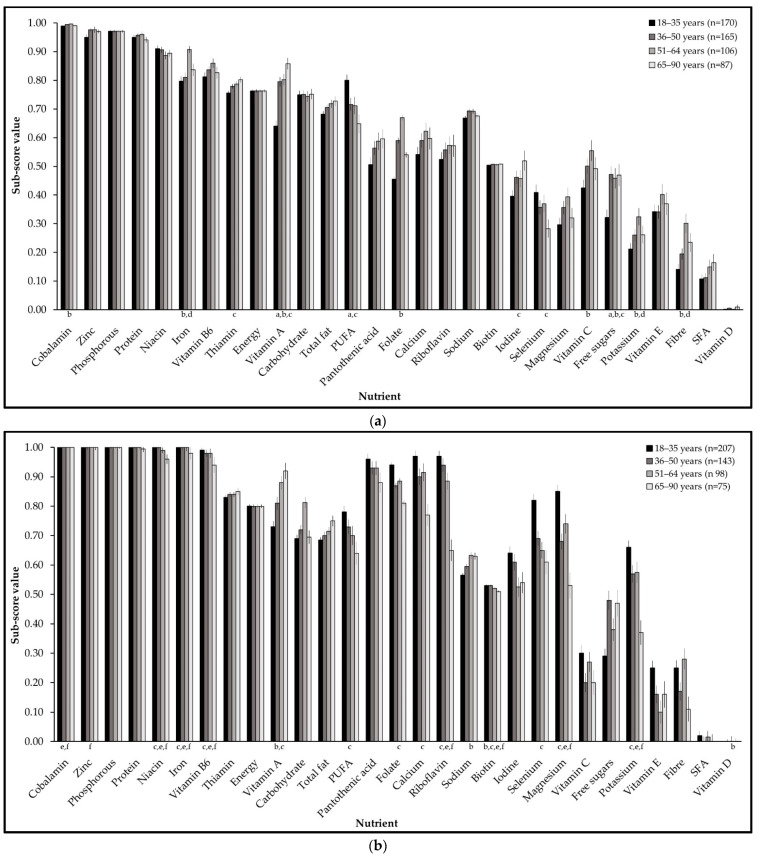
(**a**) Nutrient sub-scores for females by age group; (**b**) nutrient sub-scores for males by age group. Values shown as median ± std. error. SFA = saturated fatty acids. PUFA = polyunsaturated fatty acids. Significance from ANCOVA with Bonferroni corrections for multiple comparisons indicated as: a. between 18–35 and 36–50 age groups; b. between 18–35 and 51–64 age groups; c. 18–35 and 65–90 age groups; d. 36–50 and 51–64 age groups; e. 36–50 and 65–90 age groups; f. 51–64 and 65–90 age groups (*p* < 0.05).

For females, free sugars and vitamin A were significantly lower for the 18 to 35 age group than the older age groups. Cobalamin, fibre, folate, potassium, vitamin C, and iron were significantly lower for the 18 to 35 group than the 51 to 64 group. Scores for iodine and thiamin were significantly lower, but selenium and PUFA were significantly higher, for the 18 to 35 group than the 65 to 90 group. PUFA was the only sub-score significantly higher for the 18–35 age group than the 36–50 group. Fibre, iron, and potassium were significantly lower for the 36 to 50 group than the 51 to 64 age group (Figure 3).

For males, the 65 to 90 age group had significantly lower scores than the younger age groups for biotin, iron, niacin, potassium, riboflavin, and vitamin B6. Biotin, vitamin D, and sodium were significantly lower for the 18 to 35 age group than the 51–64 age group only; calcium, cobalamin, folate, and PUFA were significantly lower for the 18 to 35 group than the 65–90 group; and vitamin A was significantly lower for the 18–35 age group than the 51 to 64 and 65 to 90 age groups, but not the 36 to 50 age group (Figure 3).

### 3.5. Nutritional Adequacy and Food Group Intake at the Population Level

**Figure 4:**
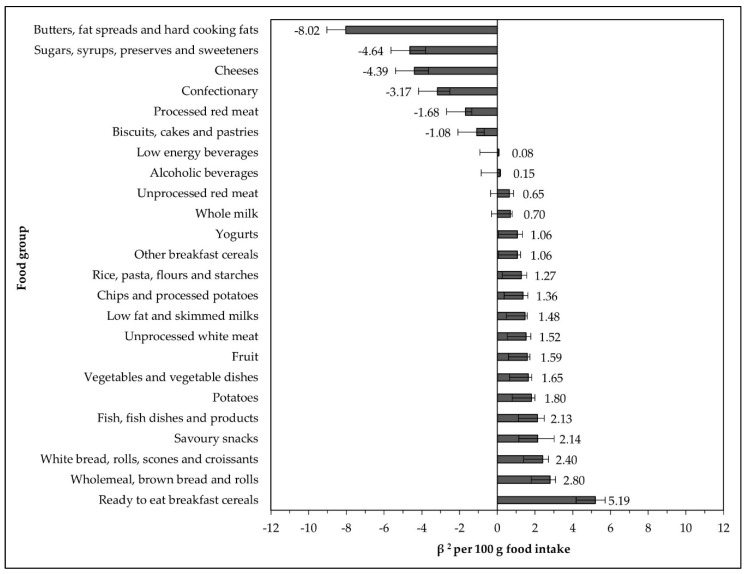
Food group intakes with a significant correlation to PANDiet scores at the population level. Multiple linear regression coefficients corrected for gender, smoking status, and education level. Residual standard error 4.3, error rate 6.8%. R_2_ = 0.67, adjusted R_2_ = 0.66 F (36 and 992) = 56.67 (*p* < 0.0001). Creams, ice-creams, rice puddings, and custard (β^2^ −0.35); eggs and egg dishes (β^2^ 0.51); fruit juices and smoothies (β^2^ 0.22); high-energy beverages (β^2^ −0.01); low-fat spreads and oils (β^2^ 1.05); other milks and milk-based beverages (β^2^ 0.39); processed white meat (β^2^ 0.5); savouries (β^2^ 0.23); and soups, sauces, and condiments (β^2^ 0.16) were not significantly correlated to PANDiet scores (*p* > 0.05).

At the population level, a significant positive relationship was found with eighteen food groups: ready-to-eat breakfast cereals (RTEBC); wholemeal/brown bread and rolls; white bread, rolls, scones, and croissants; savoury snacks; fish, fish dishes, and fish products; potatoes; vegetables and vegetable dishes; fruit; unprocessed white meat; low-fat and skimmed milks; chips and processed potatoes; rice, pasta, flours, and starches; other breakfast cereals; yogurts; whole milk; unprocessed red meat; alcoholic beverages; and low energy beverages (all *p* < 0.05). A significant negative relationship with six food groups was found, namely: butters, fat spreads, and hard cooking fats; sugars, syrups, preserves, and sweeteners; cheeses; confectionary; processed red meats; and biscuits, cakes, and pastries (all *p* < 0.05). Nine food groups did not have a significant relationship with PANDiet scores: creams, ice-creams, rice puddings, and custard; eggs and egg dishes; fruit juices and smoothies; high-energy beverages; low-fat spreads and oils; other milks and milk-based beverages; processed white meat; savouries; and soups, sauces, and condiments (*p* > 0.05) (Figure 4).

### 3.6. Food Group Intake Grouped by Age Group and Gender

The intakes of twenty-one food groups significantly differed across age groups for males and females (Table 3). For females, five food groups differed across age groups: butters, fat spreads, and hard cooking fats; cheeses; low-energy beverages; low-fat and skimmed milks; and unprocessed red meat. For males, four food groups differed across age groups: creams, ice-creams, rice puddings, and custard; fish, fish dishes, and fish products; RTEBC; and whole milk. Four food groups were not significantly different across any age group or gender: eggs and egg dishes; other milks and milk-based beverages; soups, sauces, and condiments; and white bread, rolls, scones, and croissants.

Further investigations into the significantly lower PANDiet scores in the young female group (<35 years) showed significantly higher intakes of alcoholic beverages, high-energy beverages, chips and processed potatoes, savouries, and confectionary, and lower intakes of fruit; potatoes; and wholemeal/brown breads and rolls compared with all three older age groups (Table 3). For the older male age group (>65 years), significantly higher intakes of creams, ice-creams, rice puddings, and custard; low-fat spreads and oils; other breakfast cereals; savoury snacks; sugars, syrups, preserves, and sweeteners; unprocessed white meat; and significantly lower intakes of chips and processed potatoes; confectionary; high-energy beverages; potatoes; and RTEBC were observed (Table 3).

### 3.7. Food Group Intake Grouped by Nutritional Adequacy

Food group intakes by gender and nutritional status group are outlined in Table 4. Between the low, moderate, and high nutritional adequacy groups, no significant differences were found for either gender for biscuits, cakes, and pastries; cheeses; creams, ice-creams, rice puddings, and custard; eggs and egg dishes; other milks and milk-based beverages; soups, sauces, and condiments; unprocessed red meat; or whole milk. For both males and females, only three food groups were significantly higher from low to moderate to high groups: fruit; wholemeal/brown bread and rolls; and yogurts. RTEBC was significantly higher across the three PS groups for males.

Between the low and high groups, significantly higher intakes of unprocessed white meats, and lower intakes of white bread, rolls, scones, and croissants were found for both genders. For females only, lower intakes of confectionary; low-fat oils and spreads; potatoes; savouries; and sugars, syrups, preserves, and sweeteners were observed, and for males, only significantly higher intakes of low-energy beverages were found. Food group intake, which significantly differed between low and moderate groups, but not moderate and high groups, included higher intakes of fish, fish dishes, and fish products for both genders; lower intakes of high-energy beverages, and higher intakes of low-fat and skimmed milks for females; and decreased intakes of sugars, syrups, preserves, and sweeteners, and increased intakes of low-fat spreads and oils and rice, pasta, flours, and starches for males only.

Only one food group, vegetable and vegetable dishes, was significantly higher for the high PS group for both genders. Increased intakes of low-energy beverages; low-fat and skimmed milks; rice, pasta, flours, and starches; and RTEBC were observed for females. Increased intakes of potatoes and low-fat spreads and oils, and decreased intakes of high-energy beverages were observed for males for the high PS groups vs. the low and moderate groups. Savouries were significantly lower for males between the moderate and high PS groups only.

## 4. Discussion

This is the first analysis of Irish adult food consumption data to examine nutritional adequacy using a scoring system, and to perform detailed analysis on 30 nutrients across the population and for specific population sub-groups. PANDiet scores indicate that the average adult in Ireland had a 64% probability of meeting recommendations for nutrients, without risk of excess intakes. This ranged from 38% probability to 90% for individuals. The association found between PANDiet score, gender, and education status indicate that these may be important factors to consider when addressing dietary adequacy in the Irish adult population.

The PANDiet scores identified eight nutrients of concern in the Irish adult population: vitamin D and SFA with less than 25% probability of adequacy; and a further five nutrients with less than 50% probability of adequacy, fibre, vitamin E, potassium, free sugars, and vitamin C. That said, vitamin E and potassium were assessed using the adequate intake (AI) recommendation from EFSA, and, therefore, interpretation of these results is limited. Eleven nutrients had between 50% and 75% probability of adequacy: magnesium, iodine, biotin, sodium, cobalamin, calcium, folate, pantothenic acid, selenium, PUFA, fat, and carbohydrate; and ten were seen to be high, at over 75%: vitamin A, thiamin, total energy, folate, iron, niacin, protein, zinc, phosphorus, and cobalamin. These nutrients may represent nutrients with less stable intakes across the population, and may represent nutrients to be monitored within population sub-groups. These findings were seen to be accurate relative to previous NANS findings [41] and the PANDiet scoring system, and could, therefore, be considered a valid metric for assessing nutritional adequacy in Irish food consumption databases.

The average PANDiet score was similar to that found in France, the Netherlands, and the US, with the mean PANDiet score for all countries within 5% of each other (Ireland, 63.69; France, 63.25; Netherlands, 61.10; US, 58.73) despite having slightly different nutrients and nutrient constraints [10,12]. Unlike in the France and US datasets [12], all nutrient sub-scores, apart from PUFA, were found to be significantly related to the final PANDiet scores in Ireland. This is important, as the PANDiet score should be representative of all nutrient sub-scores to avoid the oversight of nutrients. The Irish PANDiet scores are, therefore, reflective of 28 out of 29 nutrients assessed. In Ireland, females between the ages of 18 and 35 years had the lowest probability of meeting recommendations, followed by females aged between 36 and 50, and older males between the ages of 65 and 90. These population sub-groups have been highlighted as concerns for nutrient deficiencies in the Irish population in previous research [36,37]. Though males were found to have higher PANDiet scores in Ireland, this was not found in France, where older participants were found to have higher scores, and no relationship was found with gender, or in the US, where females were found to have higher scores, but no relationship with age was found. Nutritional adequacy and at-risk subgroups may, therefore, vary between countries, and should be researched at a national level. Mean sub-score values were not available for France or the US [12]; therefore, a detailed comparison of nutritional adequacy was limited.

Food groups across France, Ireland, and the US [12] that promoted probability included breakfast cereals; dairy products; fish and fish dishes; fruits; and vegetables. Intakes of unprocessed red and white meats were found to be associated with higher nutritional adequacy in the Irish population, which was not found in the France or US databases [12]. The results of the current study indicate that grouping meats into processed and unprocessed varieties may be insufficient, and red and white meats should be evaluated separately. Food groups that similarly reduced nutritional adequacy across the USA, France, and Ireland included cheeses and butters. Interestingly, potatoes were deemed a source of nutrition in the Irish and French databases, but not in the US, where a negative relationship was found, and biscuits were negatively associated with PANDiet scores in Ireland and the US, but not in France. Chips and processed potatoes were significantly associated with PANDiet scores in the Irish population, which may present a source of carbohydrates in the diet, for which over 50% of the population in NANS had below the recommended 45% of total energy. This could result from inconsistent food groupings, or a variation of nutritional quality of foods across different agricultural practices and/or markets. The nutritional value of foods should, therefore, be assessed at a national level, and more consistent food groupings should be used.

The findings of this study reinforce the nutritional adequacy of food-based dietary guidelines at a population level established in Ireland in 2011, and support the promotion of intakes of fruit; low-fat and skimmed milks; wholemeal breads and rolls; and unprocessed white meats; and decreased intakes of white bread, rolls, scones, and croissants for both genders. Intakes of low-energy-dense foods were associated with a higher probability of meeting recommendations, which is reflective of previous research relating these foods to optimal health [52]. The analysis described in the present study between low, moderate, and high nutritional status groups may provide a scientific basis for more specific guidelines based on existing diets, i.e., guidance for more “transitional” dietary changes. For example, though intakes of seventeen food groups were significantly different between low and high nutritional status groups, only three were different between low and moderate status groups for both genders. This demonstrates the varying role food groups can play in nutritional status for females and males, depending on current dietary habits. Specific guidance for specific sub-groups may improve nutrient intakes, particularly in those who at a critical stage for nutrient intakes. Specific FBDG were recently published in Ireland for older adults and young children by the Food Safety Authority of Ireland (FSAI) [53,54]. The findings of the current study support the development of more targeted food-based dietary guidelines, and indicate an urgent need for specific recommendations for younger women in Ireland. More detailed analyses should be completed to achieve this.

PANDiet scores were assessed against the Alternate Healthy Eating Index (AHEI) and Alternate Mediterranean Diet (aMED) food-based scores. Although the PANDiet scores were significantly related to the Alternative Healthy Eating Index (AHEI) and Alternate Mediterranean Diet score (aMED), this correlation was considered moderate, and, therefore, differences between the scoring systems exist. Though scoring systems such as the AHEI and aMED assess food group intakes associated with long-term health, these systems may overlook essential nutrients, as they are based on intakes of a limited number of nutrients and/or food groups. Food-based guidelines and, subsequently, scoring systems based on adherence to food intakes, do not always ensure adequacy intake of a number of nutrients, such as potassium or vitamin E [55], which were identified in this study as nutrients of concern. For example, the AHEI score does not assess dairy intake, which is an important source of iodine intakes in the Irish population [37,56]; therefore, this nutrient may be neglected in this scoring system. Moreover, food-based scoring systems, such as AHEI and aMED, may not be inclusive to individuals following specialised diets, as they assess diet quality based on a limited number of traditional food groups. Scoring systems focused on nutrient intakes, regardless of food source, may constitute a more comprehensive and objective basis for future guidelines, as individuals are not penalised for omitting food groups.

In addition to this, the PANDiet scoring system considers nutrient intakes from new products on the market that may not necessarily fit into traditional food groupings. The PANDiet scoring system avoids the complexities of aligning foods from different markets, composite dishes across countries and cultures, and assigning hybrid foods to a single food group. The PANDiet scores may, therefore, provide a more flexible approach to assessing dietary patterns, and monitoring dietary changes in the future. This monitoring may be of particular importance, as even minor dietary changes between meat types has been shown to increase iron deficiency anaemia in certain countries [57], and there is a risk that traditional sources of nutrients may be replaced with food alternatives, which are not considered nutritionally interchangeable [58]. This study supports prioritising the continued monitoring of diets to ensure that sub-groups of the population are nutritionally sufficient, and to mitigate the impact of dietary changes on nutritional adequacy, which have been shown to change in Ireland, even over short time periods [35].

PANDiet scores have been used to provide tailored dietary advice at the individual level. Studies have shown that there is sufficient evidence to support the adaption of diet quality tools in clinical dietetic practice and diet self-assessments [5], and that the PANDiet represents a scoring system for these applications [10,27,28,29]. The PANDiet system has the potential to function as a metric use in nutrition software and has been used as a basis for producing personalised dietary advice for a population sub-group in France [27]. As some countries have moved towards electronic food consumption data collection [59], the PANDiet scoring system may provide a basis for automating feedback to individuals.

The limitations of this study should also be considered. It is important to note that this study evaluated nutrient intakes from dietary sources only and did not consider intakes from nutritional supplements. This is of particular importance relative to vitamin D intakes, as there is a consensus that it is unrealistic that the Irish population can achieve vitamin D intakes from diet alone without food fortification [60,61]. In addition to this, though adequacy was low across both genders and age groups for vitamin E (<40%), supplementation has been shown to constitute over 29% of vitamin E intakes in Irish adults, and this was not considered in the current study [62]. Limitations relating to food consumption data collection are also well-established [63], and this may be reflected in the PANDiet scores [12]. Although under-reporters were removed to mitigate misreporting in the current analysis, over-reporting was not considered. The NANS data was collected over a decade ago, and more detailed analyses on dietary patterns and up-to-date dietary data would provide more relevant insight into Irish diets today. Significant differences in intakes were also only analysed for reported amounts; therefore, the importance of food groups in the Irish diet may be overlooked where intakes are currently adequate across all population sub-groups. Caution is also warranted when ascribing a single measure to nutritional adequacy, as there is a concern that this may pose a risk when interpreted by disciplines outside of nutrition. PANDiet sub-scores should be provided to mitigate this risk.

Furthermore, inaccuracies in calculating nutrient scores may exist in more complicated scoring systems, such as the PANDiet. As calculations were based on AI recommendations for some nutrients, including cobalamin, iodine, and selenium, the accuracy of the adequacy score is reduced for these nutrients, and inadequacy may be over-estimated. The PANDiet score for these nutrients should, therefore, be considered as conservative. Unfortunately, some nutrients could not be included in the scores despite nutritional recommendations existing, due to a lack of data in the food composition databases. In addition to this, parameters such as clinical status and health status were not considered, which has been recommended when evaluating dietary adequacy [48]. Scores with more components may make it more difficult to elucidate specific nutrient deficiencies in a population and are not intended to replace more detailed nutritional analyses. In-depth analyses remain important in evaluating nutritional adequacy in a population, and to capture intrinsic characteristics of food choice and behaviours.

A main concern with DQIs is the lack of robust validation and evaluation. The PANDiet scores were assessed in a subset of NANS with ten biomarkers of nutritional status. Serum folate was positively correlated with PANDiet scores in Ireland, similar to that observed with PANDiet scores in France and the US [12]. The correlation of PANDiet scores with riboflavin status and serum vitamin D in Ireland also suggests alignment of this scoring system with biomarkers of nutritional status. The PANDiet scores did not align with the remaining biomarkers of nutritional status. This may be due to the PANDiet scores considering the reporting period and calculation of usual intakes, whereas nutritional biomarkers may be reflective of more short-term intakes. The dietary data may also not match biomarker data, as discretionary use of minerals, such as sodium, may not be fully accounted for. In addition to this, additional biomarkers are required to ensure a comprehensive evaluation of PANDiet scores, which should be considered in future validation studies. For example, alpha-carotene and beta-carotene, used as validation biomarkers for the French database, were not available in the Irish or US databases. A repeated analysis of PANDiet scores on a more recent food consumption database in Irish adults, with a wider range of more stable biomarkers, would facilitate a stronger validation.

Strengths of this study include the use of probabilistic calculations of nutrient adequacy and detailed nutrient intakes, which strengthen the accuracy of the PANDiet scoring system. The PANDiet scoring system incorporates all available knowledge on individuals and nutrients, including anthropometric measurements, the reporting period, mean intake, day-to-day variability of intake, nutrient reference values, and interindividual variability, which add specificity to the score. As nutrient-based scores were calculated specifically to individual nutrient requirements, as they consider age, gender, energy intake, and/or weight, as opposed to assessing diet quality on generic FBDG, the under- and over-estimation of nutritional adequacy may be reduced.

As PANDiet scores are based on age- and sex-specific nutrient recommendations, and take anthropometric measurements into account, the PANDiet scoring system may constitute a valuable metric for assessing nutrient adequacy in Ireland across population groups. The expansion of the PANDiet scores to additional population groups using databases on children and teenagers [64,65] may facilitate cross-survey analysis, as it eliminates the lack of specificity in FBDG, and the complexities of recommended portion sizes. This would be useful in identifying nutrients of concern following dietary trends across age groups. The PANDiet scoring system could form a scientific basis for sustainable dietary guidelines when combined with environmental factors and/or dietary cost, as has been researched in France [16,17,18]. The addition of a total energy sub-score in this study may promote healthier diets relative to specific energy requirements, and reduce the promotion of nutritionally adequate, but energy-incompliant, diets. The inclusion of total energy in this scoring system may strengthen the use of PANDiet scores in sustainable diet research, as energy over-consumption is associated with an increased climatic impact of food consumption [66]. As it has been recommended that sustainable dietary guidelines are developed at a national level [67], the development of new guidelines present an opportunity to provide more transitional steps towards nutritional adequacy with a reduced environmental impact, specific to population sub-groups.

As international projects move towards standardised and harmonised food consumption databases [30,31,68], nutrient-based scoring systems offer a way of evaluating and comparing diets across different countries. Though dietary guidelines can vary between countries, nutrient scoring systems use international nutrient reference values as constraints, meaning that nutritional status and diets could be compared across countries. Nutrient-based scoring systems may, therefore, improve the interoperability and compatibility of datasets within food consumption databases, and could facilitate a cross-comparison of dietary patterns internationally.

In conclusion, the PANDiet score represents a new method of assessing dietary quality in the Irish food consumption databases and may provide a progressive method of monitoring nutrient sources in Ireland as dietary patterns change. This scoring system is deemed comprehensive and reflective of dietary quality across population sub-groups and may prove a valuable metric for inter-disciplinary research in the future, in addition to facilitating a cross-country comparison of diets.

## Figures and Tables

**Table 1:** Overview of nutrient sub-scores, and correlation with PANDiet score and nutrient sub-scores and food-based DQIs.

Nutrient Sub-Score	Mean Score	±SEM	Q1	Q2	Q3	Q4	R_s_	*p*
Energy	0.76	0.01	0.60	0.80	0.80	1.00	0.23	<0.001
Carbohydrate	0.74	0.01	0.54	0.74	0.93	1.00	0.29	<0.001
Fibre	0.28	0.01	0.02	0.12	0.48	1.00	0.62	<0.001
Free sugars	0.42	0.01	0.04	0.37	0.79	1.00	0.23	<0.001
Fat	0.71	0.00	0.99	1.00	1.00	1.00	0.50	<0.001
PUFA	0.70	0.01	0.46	0.80	0.98	1.00	−0.06	<0.001
SFA	0.12	0.01	0.00	0.01	0.11	1.00	0.43	0.05
Protein	0.96	0.00	0.98	1.00	1.00	1.00	0.40	<0.001
Vitamin A	0.75	0.01	0.56	0.82	0.97	1.00	0.31	<0.001
Thiamin	0.80	0.00	0.72	0.80	0.88	1.00	0.57	<0.001
Riboflavin	0.67	0.01	0.38	0.79	0.98	1.00	0.55	<0.001
Niacin	0.93	0.00	0.91	0.98	1.00	1.00	0.40	<0.001
Pantothenic acid	0.68	0.01	0.42	0.77	0.97	1.00	0.52	<0.001
Vitamin B6	0.87	0.01	0.81	0.95	1.00	1.00	0.56	<0.001
Biotin	0.52	0.00	0.49	0.51	0.54	0.67	0.55	<0.001
Folate	0.68	0.01	0.44	0.76	0.98	1.00	0.61	<0.001
Cobalamin	0.99	0.00	1.00	1.00	1.00	1.00	0.49	<0.001
Vitamin C	0.44	0.01	0.08	0.35	0.84	1.00	0.45	<0.001
Vitamin D	0.01	0.00	0.00	0.00	0.00	0.99	0.10	<0.001
Vitamin E	0.35	0.01	0.04	0.22	0.64	1.00	0.37	<0.001
Calcium	0.68	0.01	0.40	0.79	0.98	1.00	0.46	<0.001
Iodine	0.52	0.01	0.22	0.50	0.82	1.00	0.44	<0.001
Iron	0.89	0.01	0.85	0.98	1.00	1.00	0.49	<0.001
Magnesium	0.50	0.01	0.15	0.47	0.87	1.00	0.62	<0.001
Phosphorous	0.97	0.00	0.99	1.00	1.00	1.00	0.42	<0.001
Potassium	0.41	0.01	0.07	0.32	0.72	1.00	0.60	<0.001
Selenium	0.51	0.01	0.17	0.50	0.85	1.00	0.32	<0.001
Sodium	0.65	0.00	0.55	0.66	0.75	0.78	0.14	<0.001
Zinc	0.98	0.00	0.99	1.00	1.00	1.00	0.42	<0.001
Adequacy sub-score	66.59	0.41	60.85	72.22	81.89	97.30	0.71	<0.001
Moderation sub-score	60.78	0.32	50.00	57.67	65.00	94.83	0.46	<0.001
PANDiet score	63.69	0.23	59.21	64.34	69.63	94.74	-	-

Abbreviations: PUFA = polyunsaturated fatty acids. SEM = standard error of the mean. SFA = saturated fatty acids. R_s_ = Spearman’s rank correlation coefficient. Significance considered at the 0.05 level (*p*). There was an independence of residuals, as assessed by a Durbin–Watson statistic of 0.192 (*p* = 0.086). Q1–Q4 indicate 25% quantiles (25–100%).

**Table 2:** Linear regression analysis of the PANDiet score with demographic and biofluid factors.

	β^2^ (5–95% CI)	*p*
Demographic factors (*n* = 1051)		
Age (years)	0.03 ± 0.02 (0.00, 0.06)	0.11
Gender (female)	−4.07 ± 0.64 (−5.12, −3.02)	<0.001
Education status	0.73 ± 0.26 (0.30, 1.17)	<0.01
Social class	−0.10 ± 0.21 (−0.44, 0.25)	0.65
Smoker	1.07 ± 0.26 (0.63, 1.50)	<0.001
Body fat (%)	0.01 ± 0.04 (−0.05, 0.07)	0.81
Energy density (g/kcal)	−11.87 ± 0.75 (−13.11, −10.63)	<0.001
Biomarkers (*n* = 771)		
Serum calcium	−1.36 ± 1.41 (−3.67, 0.96)	0.34
Serum ferritin	0.00 ± 0.00 (−0.01, 0.00)	0.10
Riboflavin status	−4.18 ± 1.29 (−6.30, −2.06)	<0.01
Vitamin B6 status	0.00 ± 0.00 (0, 0.01)	0.25
Serum folate	0.04 ± 0.01 (0.02, 0.06)	<0.001
Serum vitamin B12	0.00 ± 0.00 (0.00, 0.00)	0.39
Vitamin D (s25OHD)	0.02 ± 0.01 (0.00, 0.03)	<0.05
Urinary creatinine	0.00 ± 0.00 (0.00, 0.00)	0.51
Urinary potassium	0.01 ± 0.01 (−0.01, 0.02)	0.53
Urinary sodium	0.00 ± 0.01 (−0.01, 0.01)	0.64

Abbreviations: β^2^ = beta coefficient. CI = confidence interval. *p* = *p*-value. <0.05 considered significant. All variables are corrected for nutritional supplement intake and significant factors. Residual standard error 5.16, error rate 8%. R_2_ = 0.48, adjusted R_2_ = 0.47 F (18 and 618) = 47.71 (*p* < 0.0001).

**Table 3:** Food group intakes by age group and gender.

Age Group (Years)	Females				Males			
	18–35 *n* = 170	36–50 *n* = 165	51–64 *n* = 106	65–90 *n* = 87	18–35 *n* = 207	36–50 *n* = 143	51–64 *n* = 98	65–90 *n* = 75
Food Group (grams per day)								
Alcoholic beverages ^†,‡^	782 ± 952 ^a^	484 ± 662 ^b^	390 ± 612 ^b,c^	243 ± 469 ^c^	266 ± 380 ^a^	169 ± 261 ^b^	106 ± 162 ^b^	33.2 ± 69.6 ^b^
Biscuits, cakes, and pastries ^†,‡^	28.2 ± 39.8 ^a^	39.8 ± 40.9 ^b^	52.7 ± 53.7 ^b^	39.1 ± 42.6	24.9 ± 26.9 ^a^	35.1 ± 32.1	39.0 ± 34.9 ^b^	30.3 ± 28.4
Butters, fat spreads, and hard cooking fats ^†,‡^	12.5 ± 13.6 ^a^	15.2 ± 17.0	17.7 ± 19.8	18.9 ± 28.4 ^b^	7.42 ± 9.7	10.6 ± 10.3	10.7 ± 16.2	14.0 ± 19.0
Cheeses ^†,‡^	20.1 ± 22.8 ^a^	18.1 ± 24.1 ^b^	14.0 ± 16.5	16.6 ± 23.1 ^b^	15.8 ± 16.9	11.4 ± 12.9	11.5 ± 14.9	7.73 ± 10.7
Chips and processed potatoes ^†,‡^	70.4 ± 71.8 ^a^	62.1 ± 58.7 ^b^	46.1 ± 52.5 ^b,c^	29.0 ± 43.2 ^c^	58.6 ± 56.3 ^a^	44.8 ± 49.4 ^a^	36.4 ± 39.1	20.3 ± 34.4 ^b^
Confectionary ^†,‡^	22.6 ± 31.2 ^a^	14.1 ± 19.8 ^b^	11.4 ± 18.0 ^b,c^	3.77 ± 9.9 ^c^	21.4 ± 22.5 ^a^	13.6 ± 16.9 ^b^	11.7 ± 15.3 ^b,c^	6.02 ± 11.0 ^c^
Creams, ice-creams, rice puddings, and custard	15.8 ± 34.9	18.1 ± 29.2	30.6 ± 46.4	41.6 ± 55.8	22.9 ± 38.3 ^a^	23.1 ± 32.6 ^a^	24.9 ± 37.0	32.7 ± 41.6 ^b^
Eggs and egg dishes ^‡^	19.7 ± 31.1	18.1 ± 24.2	21.2 ± 22.4	22.4 ± 28.9	13.9 ± 20.2	16.2 ± 22.1	16.7 ± 23.2	14.9 ± 18.5
Fish, fish dishes, and fish products ^†,‡^	21.5 ± 36.4	30.1 ± 46.6	39.3 ± 51.9	45.6 ± 54.4	21.6 ± 32.1 ^a^	30.3 ± 36.9	32.8 ± 38.4 ^b^	33.4 ± 39.9 ^b^
Fruit ^†^	76.1 ± 100 ^a^	80.6 ± 94.6 ^b^	115 ± 120 ^b^	122 ± 148 ^b^	61.6 ± 82.7 ^a^	102 ± 104	175 ± 147 ^b^	141 ± 122 ^b^
Fruit juices and smoothies ^‡^	82.1 ± 126 ^a^	55.3 ± 106	44.5 ± 81.1 ^c^	43.5 ± 67.7	62.7 ± 89.8 ^a^	45.5 ± 77.8	32.0 ± 63.4 ^b^	48.2 ± 75.9 ^b^
High-energy beverages	191 ± 210 ^a^	113 ± 198 ^b^	58.4 ± 126 ^b^	15.1 ± 43.9 ^b^	151 ± 231 ^a^	57.0 ± 131 ^b^	31.2 ± 73.9 ^b,c^	7.0 ± 24.2 ^c^
Low-energy beverages ^†,‡^	1178 ± 759 ^a^	1118 ± 627 ^b^	1090 ± 465 ^b^	1009 ± 745	1049 ± 626	1290 ± 698	1283 ± 583	1138 ± 538
Low-fat and skimmed milks ^†,‡^	88.8 ± 155 ^a^	112 ± 192	90.6 ± 159	103 ± 142 ^b^	77.6 ± 110	105 ± 148	112 ± 122	131 ± 170
Low-fat spreads and oils ^‡^	2.0 ± 4.6 ^a^	4.3 ± 10.2 ^a^	8.8 ± 17.5 ^b^	11.8 ± 20.5	2.5 ± 5.9 ^a^	3.51 ± 7.4 ^a^	7.8 ± 13.9 ^b^	5.2 ± 10.3 ^b^
Other breakfast cereals ^†^	18.2 ± 50.6 ^a^	38.3 ± 91.6 ^a^	60.0 ± 114 ^b^	82.8 ± 108 ^b^	15.0 ± 46.5 ^a^	31.8 ± 69.4 ^a^	64.8 ± 100	78.0 ± 103 ^b^
Other milks and milk-based beverages ^‡^	15.8 ± 67.9	9.9 ± 53.1	9.8 ± 44.5	6.91 ± 38.7	25.0 ± 58.4	22.6 ± 61.7	11.1 ± 39.1	18.1 ± 55.7
Potatoes ^†,‡^	72.7 ± 87.5 ^a^	89.9 ± 89.0 ^b^	124 ± 93.0 ^b,c^	43.7 ± 49.6 ^c^	42.0 ± 53.1 ^a^	64.5 ± 60.2 ^a^	78.4 ± 67.0 ^b^	20.9 ± 22.5 ^b^
Processed red meat ^†,‡^	67.5 ± 58.9 ^a^	58.9 ± 54	46.2 ± 42.9 ^b^	11.6 ± 27.6 ^b^	37.6 ± 39.4 ^a^	28.7 ± 35.3	25.5 ± 27 ^b^	6.8 ± 19.9 ^b^
Processed white meat ^‡^	30.0 ± 40.7 ^a^	13.4 ± 27.1	11.3 ± 23.8 ^b^	15.9 ± 47.5 ^b^	17.9 ± 29.2 ^a^	11.7 ± 24.7 ^b^	7.78 ± 21.2 ^b^	15.2 ± 30.5 ^b^
Ready-to-eat breakfast cereals (RTEBC) ^†^	59.3 ± 75.4	37.4 ± 47.9	31.0 ± 54.2	15.8 ± 25.2	34.3 ± 46.3 ^a^	32.0 ± 41.3 ^a^	23.2 ± 32.5	16.6 ± 22.7 ^b^
Rice, pasta, flours, and starches ^†,‡^	35.7 ± 40.8 ^a^	31.2 ± 33.3 ^a^	24.0 ± 26.8	11.8 ± 30.0 ^b^	21.7 ± 24.4 ^a^	21.1 ± 24.9 ^b^	16.6 ± 24.2 ^b^	6.9 ± 16.9 ^b^
Savouries	62.3 ± 80.5 ^a^	31.7 ± 44.1 ^b^	27.6 ± 73.6 ^b^	0.9 ± 4.2 ^b^	41.8 ± 54.5 ^b^	18.4 ± 28 ^b^	15.1 ± 29.1 ^b^	3.7 ± 12.4 ^b^
Savoury snacks ^†,‡^	16.3 ± 22.1 ^a^	9.1 ± 16.0 ^b^	5.9 ± 15.2 ^b,c^	80.8 ± 92.8 ^c^	15.1 ± 17.6 ^a,b^	9.5 ± 12.6 ^b^	6.7 ± 12.2 ^a,b^	57.9 ± 69.4 ^c^
Soups, sauces, and condiments ^‡^	68.8 ± 72.6	55.8 ± 61.0	53.7 ± 73.8	25.9 ± 29.7	59.6 ± 63.0	48.9 ± 69.8	68.3 ± 80.3	11.8 ± 12.4
Sugars, syrups, preserves, and sweeteners ^†,‡^	8.77 ± 12.5 ^a^	16.2 ± 19	19.5 ± 21.1 ^c^	103 ± 72.6	7.41 ± 11.4 ^a^	8.93 ± 12.0 ^b^	12.2 ± 20.7 ^b,c^	78.3 ± 72.1 ^c^
Unprocessed red meat ^†,‡^	89.5 ± 76.3 ^a^	98.8 ± 75.6	98.0 ± 77.5	21.3 ± 36.3 ^b^	55.6 ± 59.3	67.1 ± 61.3	74.5 ± 61.2	37.0 ± 45.9
Unprocessed white meat ^†,‡^	68.5 ± 70.6 ^a^	60.1 ± 66.8 ^b^	40.3 ± 46.7	111.0 ± 79.5	51.1 ± 50.6 ^a^	37.2 ± 45.1 ^a^	38.6 ± 48.3	128.0 ± 68.8 ^b^
Vegetables and vegetable dishes ^†,‡^	96.1 ± 82.9 ^a^	118 ± 82.9 ^b^	132 ± 65.3 ^b^	81.5 ± 73.2	102 ± 89.2 ^a^	137 ± 98.5	142 ± 88.6 ^b^	44.2 ± 45.8
White bread, rolls, scones, and croissants ^†^	77.6 ± 60.4	83.6 ± 64.3	83.7 ± 69.1	83.3 ± 113	56.3 ± 46.6	57.0 ± 45.8	47.5 ± 51.8	95.9 ± 149
Whole milk ^†^	187 ± 260 ^a^	158 ± 192	141 ± 173	75.3 ± 74 ^b^	71.8 ± 109 ^a^	94.7 ± 165	51.4 ± 96.6	56.9 ± 47.2 ^b^
Wholemeal/brown bread and rolls ^†^	55.9 ± 57.0	65.2 ± 72.1 ^b^	78.1 ± 74.6 ^b^	24.7 ± 41.4 ^b^	38.6 ± 38.0 ^a^	56.9 ± 50.3	64.6 ± 48.0 ^b^	49.3 ± 66.6
Yogurts ^†,‡^	28.6 ± 49.4 ^a^	25.2 ± 46.5 ^c^	32.1 ± 57.7 ^b^	130 ± 99 ^b,c^	27.9 ± 41.5	31.1 ± 46.9	57.3 ± 66.4	87.8 ± 58.7
Mean PANDiet score	59.9	63.3	64.9	64.1	64.7	64.7	65.7	63.5

Statistically significant differences between groups are indicated by alternating superscript letters (*p* < 0.05) using one-way ANCOVA with Bonferroni post-hoc test for multiple comparisons. ^†^ Indicates significance with PANDiet scores at the population level when corrected for gender, education, and smoking status (*p* < 0.05). ^‡^ Indicates significantly different intakes between males and females at the population level.

**Table 4:** Food group intakes across low, moderate, and high PANDiet scores spilt by genders.

	Females			Males		
	Low	Moderate	High	Low	Moderate	High
	*n* = 176	*n* = 176	*n* = 176	*n* = 174	*n* = 174	*n* = 175
Food Group (g/mL per day)						
Alcoholic beverages ^†,‡^	189 ± 344	175 ± 279	131 ± 209	576 ± 853	657 ± 863 ^a^	417 ± 601 ^b^
Biscuits, cakes, and pastries ^†,‡^	28.4 ± 31.1	34.3 ± 33.1	32.7 ± 28.3	32.7 ± 42.8	43.9 ± 50.9	36.0 ± 37.4
Butters, fat spreads, and hard cooking fats ^†,‡^	14.5 ± 18.3 ^a^	9.0 ± 9.9 ^b^	6.9 ± 8.8 ^b^	20.1 ± 23.9 ^a^	15.5 ± 16.1 ^a^	9.95 ± 12.5 ^b^
Cheeses ^†,‡^	12.0 ± 14.4	13.7 ± 15.3	10.9 ± 14.2	19.3 ± 25.1	19.2 ± 20.2	15.3 ± 21.1
Chips and processed potatoes ^†,‡^	46.9 ± 54.4	45.9 ± 49.5	37.7 ± 43.7	62.7 ± 63.3 ^a^	64.2 ± 67.8 ^a^	46.1 ± 55.9 ^b^
Confectionary ^†,‡^	16.5 ± 21.3 ^a^	15.6 ± 17.4	11.4 ± 16.6 ^b^	15.5 ± 24.4	18.1 ± 27.4	12.8 ± 21.8
Creams, ice-creams, rice puddings, and custard	20.6 ± 29.7	27.4 ± 40.1	26.9 ± 40.0	19.1 ± 37.0	20.7 ± 36.9	28.8 ± 46.5
Eggs and egg dishes ^‡^	14.5 ± 19.8	16.5 ± 23.9	15.0 ± 19.5	18.8 ± 28.1	20.8 ± 28.9	20.2 ± 25.5
Fish, fish dishes, and fish products ^†,‡^	18.1 ± 26.8 ^a^	30.1 ± 39.6 ^b^	37.3 ± 39.1 ^b^	24.0 ± 38.9 ^a^	24.2 ± 42.9 ^a^	43.6 ± 52.5 ^b^
Fruit ^†^	59.0 ± 76.7 ^a^	90.4 ± 97.1 ^b^	181 ± 138 ^c^	40.0 ± 59.1 ^a^	81.0 ± 96.1 ^b^	152 ± 136 ^c^
Fruit juices and smoothies ^‡^	46.8 ± 84.1	56.6 ± 88.0	43.0 ± 64.5	37.9 ± 80.7 ^a^	53.7 ± 91.5 ^a^	94.8 ± 133 ^b^
High-energy beverages	114 ± 212 ^a^	69.1 ± 159 ^b^	38.0 ± 87.4 ^b^	162 ± 222 ^a^	124 ± 178 ^a^	72.3 ± 149 ^b^
Low-energy beverages ^†,‡^	998 ± 606.0 ^a^	1153 ± 565 ^a^	1407 ± 666 ^b^	985 ± 593 ^a^	1115 ± 632	1261 ± 764 ^b^
Low-fat and skimmed milks ^†,‡^	42.9 ± 74.1 ^a^	94.6 ± 134.0 ^b^	168.0 ± 158 ^b^	34.2 ± 86.8 ^a^	85.3 ± 142 ^b^	173 ± 211 ^c^
Low-fat spreads and oils ^‡^	2.1 ± 6.1 ^a^	4.4 ± 10.4	6.4 ± 10.6 ^b^	2.47 ± 6.37 ^a^	4.99 ± 14.7 ^a^	8.51 ± 14.9 ^b^
Other breakfast cereals ^†^	20.5 ± 49.1 ^a^	37.9 ± 75.5 ^a^	63.6 ± 102 ^b^	33.5 ± 89.0	42.3 ± 86.5	46.6 ± 90.9
Other milks and milk-based beverages ^‡^	16.7 ± 43.8	19.4 ± 48.8	24.8 ± 71.1	10.7 ± 55.5	12.4 ± 62.5	12.3 ± 50.9
Potatoes ^†,‡^	52.1 ± 53.6 ^a^	64.7 ± 59.6	74.8 ± 68.6 ^b^	71.6 ± 73.7 ^a^	86.3 ± 87.6 ^a^	127 ± 107 ^b^
Processed red meat ^†,‡^	38.8 ± 42.9	30.4 ± 32.7	19.7 ± 19.1	73.5 ± 62.1 ^a^	58.4 ± 53.2 ^b^	41.4 ± 40.9 ^c^
Processed white meat ^‡^	14.2 ± 26.7 ^a^	11.7 ± 22.7 ^b^	10.5 ± 26.1 ^b^	24.0 ± 37.0	18.6 ± 32.1	15.4 ± 31.6
Ready-to-eat breakfast cereals (RTEBC) ^†^	11.4 ± 16.5 ^a^	16.5 ± 19.9 ^a^	31.1 ± 29.9 ^b^	14.9 ± 21.3 ^a^	28.1 ± 33.4 ^b^	45.2 ± 40.9 ^c^
Rice, pasta, flours, and starches ^†,‡^	19.5 ± 37.0 ^a^	29.3 ± 37.4 ^a^	35.8 ± 44.8 ^b^	23.7 ± 41.1 ^a^	51.8 ± 71.7 ^b^	49.8 ± 68.3 ^b^
Savouries	28.7 ± 45.1 ^a^	24.1 ± 40.7	17.2 ± 31.9 ^b^	39.7 ± 62.6	51.4 ± 83.4 ^a^	29.6 ± 51.7 ^b^
Savoury snacks ^†,‡^	9.6 ± 15.3	10.6 ± 14.2	9.16 ± 15.0	8.18 ± 14.8 ^a^	14.4 ± 21.8 ^b^	8.01 ± 17.3 ^a^
Soups, sauces, and condiments ^‡^	50.1 ± 54.0	57.2 ± 75.0	65.9 ± 78.1	60.2 ± 71.2	71.4 ± 69.8	60.9 ± 79.2
Sugars, syrups, preserves, and sweeteners ^†,‡^	11.7 ± 19.1 ^a^	9.2 ± 11.4	7.8 ± 10.3 ^b^	19.1 ± 22.9 ^a^	13.5 ± 18.2 ^b^	13.2 ± 18.5 ^b^
Unprocessed red meat ^†,‡^	61.8 ± 63.2	70.2 ± 67.2	68.2 ± 58.2	85.8 ± 70.6	102 ± 80.9	98.6 ± 74.8
Unprocessed white meat ^†,‡^	34.6 ± 38.8 ^a^	42.9 ± 48.5	48.3 ± 54.7 ^b^	44.9 ± 57.0 ^a^	53.5 ± 55.6	63.9 ± 75.5 ^b^
Vegetables and vegetable dishes ^†,‡^	94.4 ± 69.0 ^a^	116 ± 77.8 ^a^	165 ± 106.0 ^b^	88.2 ± 60.3 ^a^	107 ± 84.1 ^a^	138 ± 86.4 ^b^
White bread, rolls, scones, and croissants ^†^	61.2 ± 51.9 ^a^	51.8 ± 46.3	45.2 ± 42.7 ^b^	92.9 ± 67.9 ^a^	81.9 ± 63.7	68.1 ± 61.1 ^b^
Whole milk ^†^	78.7 ± 126	84.3 ± 148.0	73.5 ± 129.0	129 ± 145.0	179 ± 225.0	158 ± 252.0
Wholemeal/brown bread and rolls ^†^	36.7 ± 43.3 ^a^	54.1 ± 42.1 ^b^	66.8 ± 49.4 ^c^	43.3 ± 54.3 ^a^	64.5 ± 58.9 ^b^	88.3 ± 79.8 ^c^
Yogurts ^†,‡^	19.4 ± 35.7 ^a^	38.4 ± 54.4 ^b^	57.3 ± 63.4 ^c^	16.9 ± 37.7 ^a^	27.8 ± 52.3	38.5 ± 53.8 ^b^
PANDiet score μ	54.3	62.3	71.3	57.3	64.8	72.1
PANDiet score range	38.3–58.8	58.8–66.1	66.1–89.6	42.0–62.1	62.2–63.3	67.5–89.7
Age (years) μ	43.3	43.2	50.7	43.3	40.6	44.5
Age (years) range	18–87	18–90	18–82	18–86	18–88	18–82

Statistically significant differences between groups are indicated by alternating superscript letters (*p* < 0.05) using one-way ANCOVA with Bonferroni post-hoc test for multiple comparisons. ^†^ Indicates significance with PANDiet scores at the population level when corrected for gender, education, and smoking status (*p* < 0.05). ^‡^ Indicates significantly different intakes between males and females at the population level.

## Data Availability

The data presented in this study are available from the corresponding author upon reasonable request.

## Appendix A

**Table A1:** Input data and calculated values for the energy sub-score, with penalties for over- and under-consumption.

	Females (*n* = 528)	Males (*n* = 523)
Height (metres)	1.62 ± 0.07 (1.43–1.81)	1.76 ± 0.07 (1.55–1.99)
Weight (kg)	67.8 ± 12.2 (42.8–129.0)	82.6 ± 12.8 (53.1–144.0)
Basal energy expenditure (BEE)	1365 ± 122 (1057–1894)	1759 ± 165 (1356–2356)
Total energy expenditure (TEE)	1879 ± 213 (1334–3115)	2491 ± 266 (1791–3345)

Abbreviations: kg = kilogram; BEE = basal energy expenditure; TEE = total energy expenditure. Values shown as mean ± standard deviation (minimum–maximum).

**Table A2:** Nutrient reference limits used for calculating PANDiet scores.

Nutrient	CV ^a^	Lower Limit ^a^	Upper Limit ^b^
Macronutrients			
Total Energy (EI/TEE)	-	0.95	1.05
Protein (g/kg bw/day)	12%	0.66	2.20
Carbohydrate (% EIEA)	0%	45	60
Free Sugars (% EIEA)	0%	-	10 ^c^
Dietary Fibre (g/day)	10%	25	-
Total Fat (% EIEA)	0%	20	35
Polyunsaturated Fatty Acids (% EIEA)	12%	5	-
Saturated Fatty Acids (% EIEA)	10%	-	10
Micronutrients			
Vitamin A (µg RE/day)	15%	490–570 ^d^	3000
Thiamin (mg/MJ)	20%	0.072	-
Riboflavin (mg/day)	10%	1.6	-
Niacin (mg NE/MJ)	10%	1.3	900
Pantothenic acid (mg/day)	10%	5 ^e^	-
Vitamin B6 (mg/day)	10%	1.3–1.5 ^d^	25
Biotin (µg/day)	10%	40 ^e^	-
Folate (µg DFE/day)	15%	250	1000
Cobalamin (ug/day)	10%	4 ^e^	-
Vitamin C (mg/day)	10%	80–90 ^d^	500
Vitamin D (µg/day)	10%	15 ^e^	100
Vitamin E (mg/day)	10%	11–13 ^d,e^	300
Minerals			
Calcium (mg/day)	10%	750–860 ^f^	2500
Iodine (µg/day)	20%	150 ^e^	600
Iron (mg/day)	20%	7–6 ^g^	28
Magnesium (mg/day)	10%	300 ^e^	700
Phosphorus (mg/day)	10%	550 ^e^	2500
Potassium (mg/day)	10%	3500 ^e^	-
Selenium (µg/day)	10%	70 ^e^	250
Sodium (mg/day)	10%	2000 ^e^	2759
Zinc (mg/day)	10%	12.7	25

Abbreviations: CV = co-variance; EI/TEE = energy intake/total energy expenditure; g/kg bw = grams/kilograms body weight; EIEA = energy intake excluding alcohol; ug = microgram; RE = retinol equivalents; mg = micrograms; NE = niacin equivalents; MJ= megajoule; DFE= dietary folate equivalents. ^a^ EFSA nutrient summary report [49]; ^b^ [49,51]; ^c^ WHO recommendation [59]; ^d^ lower value for females, higher value for males; ^e^ indicates lower limits available as adequate intake (AI) values only; ^f^ 750 recommended for adults >25 years, 860 recommended for adults <25 years; ^g^ lower recommendation for males and post-menopausal women, higher recommendation for pre-menopausal women (cut-off age of 51 years).

**Table A3:** Energy penalties assigned.

Energy (kcal)/TEE Difference	Sub-Score Assigned	*n*
<5%	1	218
5–20%	0.80	518
20–40%	0.60	245
40–60%	0.40	49
60–80%	0.20	17
>80%	0	4

Abbreviations: TEE = total energy expenditure. *n* = number.
